# Clinical-epidemiological characteristics and survival of cases of severe acute respiratory syndrome (SARS) due to COVID-19, according to the COVID-19 vaccination schedule in Brazil, 2021-2022: a prospective study

**DOI:** 10.1590/S2237-96222023000400003.en

**Published:** 2023-12-18

**Authors:** Carlos Martins, Victor Nogueira da Cruz Silveira, Fábio Nogueira da Silva, José de Jesus Dias, Maria dos Remédios Freitas Carvalho Branco, Alcione Miranda dos Santos, Bruno Luciano Carneiro Alves de Oliveira

**Affiliations:** 1 Universidade Federal do Maranhão, Programa de Pós-Graduação em Saúde Coletiva, São Luís, MA, Brazil

**Keywords:** COVID-19, SARS-CoV-2, Survival Analysis, COVID-19 Vaccine, Cohort Studies, COVID-19, SARS-CoV-2, Análisis de Supervivencia, Vacunas contra COVID-19, Estudios de Cohortes, COVID-19, SARS-CoV-2, Análise de Sobrevida, Vacinas contra COVID-19, Estudos de Coortes

## Abstract

**Objective:**

To analyze the clinical and sociodemographic characteristics and survival of individuals with severe acute respiratory syndrome due to COVID-19 according to the COVID-19 vaccination schedule, Brazil, 2021-2022.

**Methods:**

This was a cohort study based on data from the Influenza Epidemiological Surveillance Information System; the Kaplan-Meier and Survival Tree methods were used to analyze survival.

**Results:**

Among the 559,866 hospitalized cases, a higher proportion of vaccinated individuals was found among female (15.0%), elderly people aged ≥ 80 (34.5%), people from the Southeast region (15.7%), those who did not undergo respiratory support (21.2%) and those who did progress to death (15.2%); the survival curve showed that risk of death for unvaccinated individuals was higher in all age groups (p-value < 0.001); elderly people aged ≥ 80, who did not undergo mechanical ventilation and who had a booster dose had lower risk when compared to their peers who had two doses or were unvaccinated (hazard ratio = 0.64; 95%CI 0.62;0.67).

**Conclusion:**

Lowest risk of death was found in vaccinated individuals, especially those who had two doses or a booster dose as well.

## Study contributions

**Main results:**

Prevalence was found to be high among unvaccinated individuals. Risk of death was lower among those vaccinated with a booster dose, compared to those not vaccinated, in all age groups analyzed.

**Implications for services:**

The number of hospitalizations of unvaccinated individuals with severe acute respiratory syndrome was high, which increases the demand for health services to care for these individuals.

**Perspectives:**

It is necessary to promote widespread vaccination of the entire population of Brazil, in addition to the regular provision of booster doses for the different population groups.

## RESUMEN

**Objetivo:**

Analizar las características clínicas, sociodemográficas y supervivencia de individuos con síndrome respiratoria aguda grave por COVID-19, según el esquema de vacunación contra COVID-19, Brasil, 2021-2022.

**Métodos:**

Estudio de cohorte con datos del Sistema de Informação de Vigilância Epidemiológica de la Gripe; se utilizó el método de Kaplan-Meier y el árbol de supervivencia para analizar la supervivencia.

**Resultados:**

Entre los 559.866 casos hospitalizados, se observó una mayor proporción de vacunados entre el sexo femenino (15,0%), ancianos ≥ 80 (34,5%), región Sudeste (15,7%), quienes no recibieron soporte ventilatorio (21,2%) y no fallecidos (15,2%); la curva de supervivencia mostró que los no inmunizados presentaron un mayor riesgo de óbito en todos los grupos de edad (p-valor < 0,001); los ancianos ≥ 80, que no recibieron ventilación mecánica, con dosis de refuerzo, tienen un menor riesgo en comparación con sus pares con dos dosis o no vacunados (HR = 0,64; IC_95_% 0,62;0,67).

**Conclusión:**

El menor riesgo de óbito se observó en individuos vacunados, especialmente aquellos con dos dosis o refuerzo.

## RESUMO

**Objetivo:**

Analisar as características clínicas e sociodemográficas e a sobrevida de indivíduos com síndrome respiratória aguda grave por covid-19, segundo esquema vacinal contra covid-19, Brasil, 2021-2022.

**Métodos:**

Estudo de coorte, com dados do Sistema de Informação de Vigilância Epidemiológica da Gripe; métodos de Kaplan-Meier e árvore de sobrevivência foram utilizados para analisar a sobrevida.

**Resultados:**

Dos 559.866 casos internados, observou-se maior proporção de vacinados entre sexo feminino (15,0%), idosos ≥ 80 anos (34,5%), na região Sudeste (15,7%), entre os que não receberam suporte ventilatório (21,2%) e os não evoluídos a óbito (15,2%); na curva de sobrevida, não imunizados apresentaram maior risco de óbito, independentemente da faixa etária (p-valor < 0,001); idosos que não realizaram ventilação mecânica, com dose de reforço, apresentaram menor risco, comparados a seus pares com duas doses ou não imunizados (*hazard ratio* = 0,64; IC_95_% 0,62;0,67).

**Conclusão:**

Observou-se menor risco de ocorrência de óbito nos indivíduos vacinados, especialmente com duas doses ou reforço.

## INTRODUCTION

Globally, emergency use of vaccines represented a decisive step in public health actions to control the COVID-19 pandemic. However, vaccination of the population did not occur simultaneously, between countries, having started at the end of 2020 and the first months of 2021 in middle and high income countries, and later in countries with lower income levels. A previous study demonstrated income disparity as a factor influencing inequality in the distribution and administration of vaccines around the world: middle- and low-income countries had lower vaccination coverage.^1^

In Brazil, a nation of continental dimensions with a large population, campaigns to vaccinate against COVID-19, started late, at the end of January 2021, contributing to the spread of the pandemic throughout the national territory:^2^ the scenario in the period before the start of vaccination was characterized by an unprecedented increase in the number of hospitalizations and serious cases, as well as deaths from the disease.^3^

The collaboration of international institutions enabled two COVID-19 vaccines, Coronavac [produced by the Butantan Institute (a scientific research institute)] and Oxford/AstraZeneca [produced by the Oswaldo Cruz Foundation (Fiocruz)], to be released by the National Health Surveillance Agency (*Agência Nacional de Vigilância Sanitária* - ANVISA) and the Ministry of Health, initially for emergency use;^4^ in addition to them, currently, the Comirnaty vaccine (Pfizer/Wyeth) and the Janssen vaccine (Janssen-Cilag) are authorized for use in Brazil.^5^

The World Health Organization (WHO), in turn, defined priority groups for vaccination and recommended focusing public health actions, initially, on directly reducing morbidity and mortality, maintaining essential services and protecting those with high exposure to the virus due to their providing services to the community or for specific vulnerabilities. These recommendations were adhered to in Brazil.^6^

Providing vaccination against COVID-19 resulted in a significant reduction in the transmission of the virus, including during the phase of the highest number of hospitalizations due to the disease.^7^ Vaccines have shown immunizing potential in preventing serious clinical conditions, which place heavy pressure on health systems for long periods and with high hospitalization costs, in addition to often leading to lower survival of those infected and, consequently, death.^8^

There are studies that have carried out survival analyses, comparing those vaccinated and not vaccinated against COVID-19, in different scenarios and/or specific populations. One of them examined survival of solid organ transplant recipients in the United States in 2022;^9^ another study included people with autoimmune rheumatic diseases, assisted at a Center of Excellence in Arthritis and Rheumatism in southern India between March and October 2021.^10^ Those studies^9^-^10^demonstrated that vaccination improved the immune response and increased the survival of cases. In their research carried out in the municipality of Phuentsholing, Bhutan, with individuals with COVID-19 monitored until May 2021, Gyeltshen et al.^11^ identified a 77% lower probability of developing COVID-19 symptoms among those who had been vaccinated.

In Brazil, the results of a study of data on 400,000 individuals hospitalized due to COVID-19, between January 2021 and December 2022, demonstrated that case fatality ratio increased with age and decreased with vaccination, especially after the second dose.^12^

Despite the high volume of data on the topic, the studies cited^9^-^10^ focused on specific samples that had previous comorbidities; the Brazilian studies,^12^-^14^ in particular, did not perform survival analysis in terms of time and interaction between the predictor variables. 

The objective of this study was to describe the clinical and sociodemographic characteristics and to analyze the survival of individuals with severe acute respiratory syndrome (SARS) due to COVID-19, according to the COVID-19 vaccination schedule in Brazil.

## METHODS

This was a cohort study, conducted with cases of SARS due to COVID-19 recorded on the Influenza Epidemiological Surveillance Information System (*Sistema de Informação de Vigilância Epidemiológica da Gripe* - SIVEP-Gripe). SIVEP-Gripe, created in 2000, is an influenza monitoring system in Brazil. Once community transmission of SARS-CoV-2 was declared, on March 20, 2020, SIVEP-Gripe was adapted to receive information on acute respiratory syndromes caused by SARS-CoV-2, influenza and other respiratory viruses. COVID-19 notifications are compulsory and correspond to individuals treated in public and private hospitals, and those who died without being hospitalized. These records are available on the OpenDataSUS website (https://opendatasus.saude.gov.br/). 

The sample that served as the basis for the present study consisted of cases of adults and elderly people (age ≥ 20 years) hospitalized with diagnosis of SARS due to COVID-19, notified between 2021 and 2022, in all five Brazilian macro-regions, with information on vaccination against COVID-19. Records with missing data on case progression and length of hospital stay were excluded. Records from 2020 were excluded from the analysis because in that year vaccination against COVID-19 was not yet available in Brazil. We used the database updated as at May 31, 2023.

### Variables

Survival time, counting from hospital admission to the date of death or discharge within 90 days, was obtained from information available in the database on the date of admission and case progression. Survival time (time elapsed until death occurring within 90 days) was considered the primary outcome. We only analyzed cases that died from COVID-19, according to the case progression described on the SIVEP-Gripe form. Cases with more than 90 days of hospitalization or discharge before this period were censored (right-censor). Censoring occurred at 90 days because, after this time, the cases had a similar probability of survival.

The COVID-19 vaccination schedule was categorized as follows: “not immunized” (not vaccinated or with an incomplete vaccination schedule); “two doses”; and “booster dose”. The variable was built from the database fields FAB_COV_1, DOSE_1_COV, DOSE_2_COV and DOSE_REF, which provide information about the manufacturer of the first dose and the administration date of each dose. Cases with information about the first dose of the Janssen vaccine were included in the “two doses” category. We only considered cases with information retrieved via linkage with the National Vaccination Database, so as to avoid inconsistencies in the filling out of these fields. 

The other explanatory variables were:

a) year of hospitalization (2021 and 2022);
b) sex (male; female);
c) age group (age on last birthday: 20-39; 40-59; 60-79; 80 or over);
d) race/skin color (White; Black; Asian; mixed race; Indigenous);
e) Brazilian macro-region (Southeast; South; Midwest; North; Northeast);
f) presence of risk factors/comorbidities (none; one or more);
g) hospitalized in an intensive care unit (ICU) (yes; no); and
h) respiratory support (not performed; non-invasive; invasive).

Risk factors or comorbidities were defined according to the number of pre-existing medical conditions reported on the system, namely: being in the postpartum period, having chronic cardiovascular disease, chronic hematologic disease, chronic liver disease, asthma, diabetes *mellitus*, chronic neurological disease, chronic pneumopathy, immunosuppression, chronic kidney disease and/or obesity.

### Statistical analysis

The analyses were performed using an open access statistical program, namely the R application version 4.3.0 (R Core Team, 2021), adopting a 5% significance level for all analyses.

Initially, in the pre-processing and data organization stage, a single imputation was performed using the Fully Conditional Specification (FCS) method, implemented using the Multivariate Imputation by Chained Equations (MICE) package,^15^ with the aim of correcting missing data for some variables. Then, the descriptive analysis was carried out and means and standard deviations (SD) were calculated for the numerical variables, and absolute (n) and relative frequencies (%) for the categorical variables, according to the vaccination schedule. Differences in the frequency distribution of characteristics, between the vaccinated and unvaccinated groups, were analyzed using Pearson’s chi-square test and the Mann-Whitney test.

Comparison of the case survival curves according to the vaccination schedule was carried out using the Kaplan-Meier method. The log-rank test was used to verify differences between groups. Survival curves, stratified by age, were also compared. The Survival and Survminer applications were used for these analyses. 

The survival tree method was applied to identify different groups at risk of death from COVID-19, based on interactions between the variables studied. This is a non-parametric technique that incorporates tree-structured regression models, grouping individuals according to survival time and the variables included in the model. The Survival, LTRCtrees and Party.kit applications were used to perform the survival tree method. When comparing groups, created from the terminal nodes of the survival tree, and obtaining the hazard ratio (HR), we adjusted Cox proportional hazards models.

The study project was approved by the Research Ethics Committee of the *Hospital Universitário* of the *Universidade Federal do Maranhão* and by the National Research Ethics Commission of the National Health Council: Opinion No. 4.098.427 and Certificate of Submission for Ethical Appraisal (*Certificado de Apresentação para Apreciação Ética* - CAAE) No. 32206620.0.0000.5086, issued on June 19, 2020, in compliance with National Health Council Resolution No. 466, dated December 12, 2012.

## RESULTS

**Figure 1:**
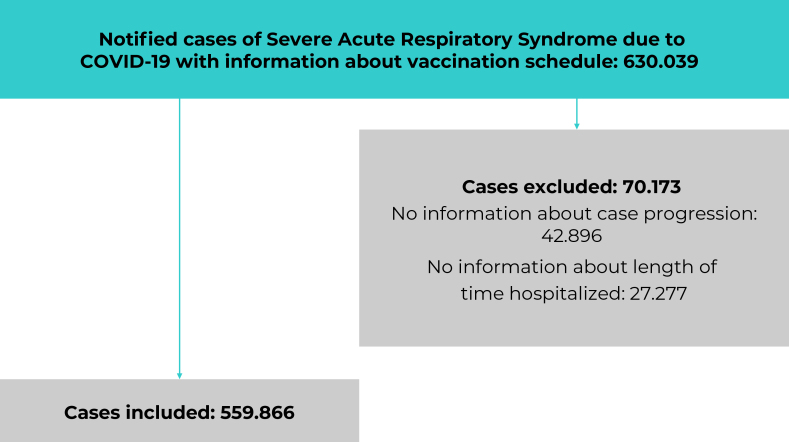
Study sample composition process after applying the exclusion criteria, Brazil, 2021-2022

Of the total 630,039 notified SARS cases that met the inclusion criteria, 70,173 (11.2%) were excluded due to lack of data on length of hospital stay and case progression, resulting in a final sample of 559,866 cases (Figure 1). Of these, 71.8% were not vaccinated or had an incomplete vaccination schedule, while 14.9% had received two doses and 13.3% had received a booster dose. In 2021, only 3.6% had been immunized with the booster dose, whereas in 2022, this percentage rose to 42.7% (Table 1)

**Table 1:** Characteristics of the individuals studied, according to vaccination schedule, Brazil, 2021-2022

Variables	Not vaccinated n (%)	Two doses n (%)	Booster n (%)	p-value^a^
**Total**	402,008 (71.8)	83,343 (14.9)	74,515 (13.3)	
**Year**				**< 0.001**
2021	363,870 (86.5)	41,866 (9.9)	15,096 (3.6)	
2022	38,138 (27.4)	41,477 (29.8)	59,419 (42.7)	
**Sex**				**< 0.001**
Male	229,599 (74.1)	43,025 (13.9)	37,109 (12.0)	
Female	172,409 (68.9)	40,318 (16.1)	37,406 (15.0)	
**Age (last birthday)**				**< 0.001**
20-39	82,949 (84.7)	9,719 (9.9)	5,317 (5.4)	
40-59	182,268 (84.5)	20,267 (9.4)	13,145 (6.1)	
60-79	108,221 (62.5)	33,983 (19.6)	30,831 (17.8)	
≥ 80	28,570 (39.0)	19,374 (26.5)	25,222 (34.5)	
**Race/skin color**				**< 0.001**
White	225,205 (70.3)	46,956 (14.7)	48,224 (15.1)	
Black	18,305 (72.4)	3,954 (15.6)	3,030 (12.0)	
Asian	4,482 (66.7)	1,055 (15.7)	1,185 (17.6)	
Mixed race	153,407 (74.3)	31,167 (15.1)	21,984 (10.6)	
Indigenous	609 (66.8)	211 (23.1)	92 (10.1)	
**Brazilian macro-region**				**< 0.001**
Southeast	201,303 (69.8)	41,828 (14.5)	45,137 (15.7)	
South	88,457 (73.6)	17,653 (14.7)	14,154 (11.8)	
Midwest	45,674 (74.2)	9,376 (15.2)	6,466 (10.5)	
North	21,578 (83.2)	3,131 (12.1)	1,211 (4.7)	
Northeast	44,996 (70.4)	11,355 (17.8)	7,547 (11.8)	
**Risk factor**				**< 0.001**
None	221,523 (77.9)	34,871 (12.3)	28,115 (9.9)	
One or more	180,485 (65.5)	48,472 (17.6)	46,400 (16.9)	
**Hospitalized in ICU** ^b^				**< 0.001**
Yes	149,618 (72.5)	32,037 (15.5)	24,787 (12.0)	
No	252,390 (71.4)	51,306 (14.5)	49,728 (14.1)	
**Respiratory support**				**< 0.001**
Not performed	62,568 (59.4)	20,436 (19.4)	22,307 (21.2)	
Non-invasive	247,270 (73.2)	47,831 (14.2)	42,631 (12.6)	
Invasive	92,170 (78.9)	15,076 (12.9)	9,577 (8.2)	
**Length of time hospitalized**				**< 0.001**
Mean (standard deviation)	11.7 (12.3)	12.1 (13.7)	11.3 (13.2)	
Median (Q1;Q3)	8.0 (4.0;14.0)	8.0 (4.0;15.0)	7.0 (4.0;13.0)	
**Death**				**< 0.001**
No	267,308 (69.5)	58,878 (15.3)	58,305 (15.2)	
Yes	134,700 (76.8)	24,465 (14.0)	16,210 (9.2)	

a) Pearson’s chi-square test, for the qualitative variables; and Mann-Whitney test, for the quantitative variables; b) ICU = Intensive Care Unit.

Regarding the sex of the cases, 15.0% of females and 12.0% of males (p-value < 0.001) had been vaccinated with a booster dose. Regarding age group, among the elderly, aged 60 to 79 and 80 years or more, 17.8% and 34.5% had received a booster dose, respectively. Percentage vaccination with a booster dose, among cases of Black, mixed race and Indigenous race/skin color – 12.0%, 10.6% and 10.1%, respectively, – was lower than that of those who were not immunized or who had received only one dose (p-value < 0.001). In the Northern region only 4.7% of cases had received a booster dose (Table 1).

Lower adherence to the booster dose was identified among those who did not have serious COVID-19 risk factors (9.9%), those admitted to an ICU (12.0%), those who required invasive respiratory support (8.2%) and those who died (9.2%) (p-value < 0.001) (Table 1).

**Figure 2:**
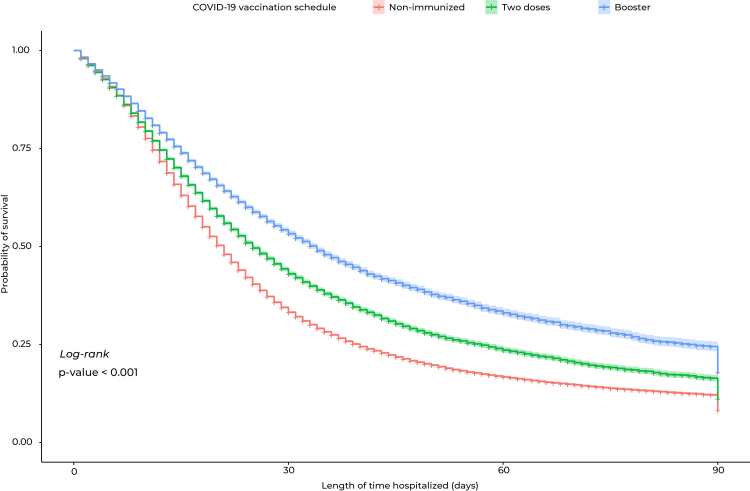
Kaplan-Meier survival curve for the individuals studied, according to vaccination schedule, Brazil, 2021-2022

**Figure 3:**
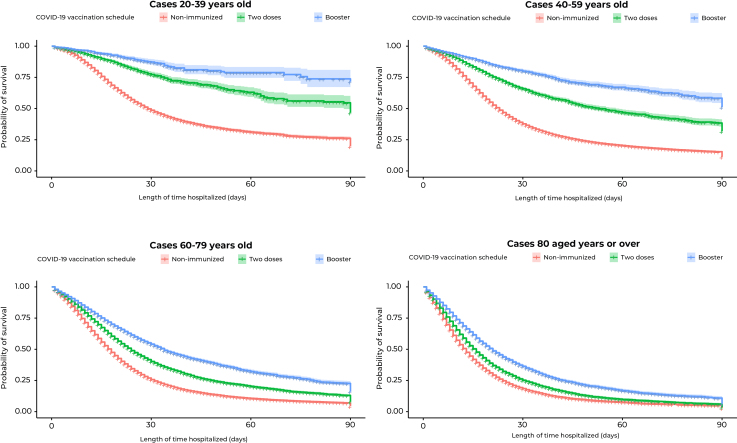
Kaplan-Meier survival curve for the individuals studied, by age group, according to vaccination schedule, Brazil, 2021-2022

The hospital survival curve over the 90-day period showed that non-immunized individuals had lower survival rates when compared to those vaccinated with two doses or those with who had had a booster dose, showing statistical difference (p-value < 0.001) (Figure 2). When analyzing the survival curves by age group, lower survival was found for non-immunized elderly people, compared to those vaccinated with two doses or with a booster dose as well (p-value < 0.001) (Figure 3).

**Figure 4:**
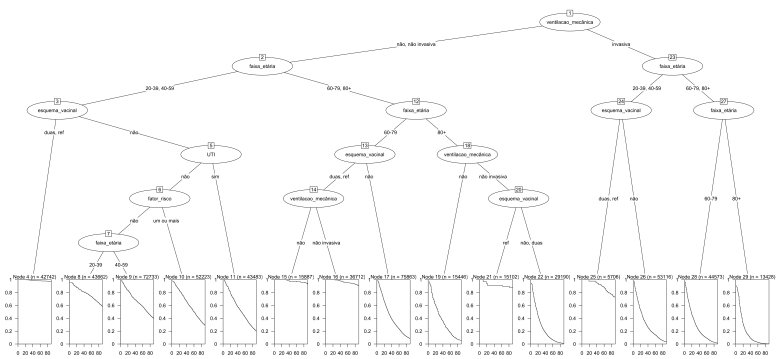
Survival tree of individuals studied, Brazil, 2021-2022

The survival tree we created identified 15 terminal nodes, based on the interaction between mechanical ventilation, age group, risk factor, ICU admission and vaccination schedule (Figure 4). Based on the Kaplan-Meier curve plotted at each terminal node, we found that the group with the lowest risk of death was that which had received at least two doses of vaccine: nodes 4, 15, 16, 21 and 24. The analysis identified the elderly as a risk group for death from COVID-19. The survival tree also demonstrated that those aged 80 or over, who did not undergo mechanical ventilation and who were vaccinated with a booster dose (node ​​21), were at lower risk of death, when compared to their peers who had had two vaccine doses or who had not been immunized (node ​​22) [hazard ratio (HR) = 0.64; 95%CI 0.62;0.67]. Similarly, cases up to 59 years of age, who had had invasive mechanical ventilation and received two doses or a booster dose as well (node ​​25) showed greater survival when compared to non-immunized individuals in the same age group (node ​​26) (HR = 0.62; 95%CI 0.60;1.64). 

1,14,18) Ventilação mecânica: respiratory support (not performed; noninvasive; invasive); 2,7,12,23,27) Faixa etária: age group (age on last birthday: 20-39; 40-59; 60-79; 80 or over); 3,13,20,24) Esquema vacinal: vaccination schedule [not immunized (not vaccinated or with an incomplete vaccination schedule); two doses; and booster dose]; 5) UTI: hospitalized in an intensive care unit [ICU, (yes; no)]; 6) Fator de risco: presence of risk factors/comorbidities (none; one or more). Notes: The squares represent the terminal nodes; each one contains the number of cases in that node (n) and the survival curve, estimated by the Kaplan-Meier method. The circles represent the internal nodes (intermediate or decision-making), contemplating the decisionmaking explanatory variable that will lead to a new split of the tree. Vaccination schedule: “no” for not immunized; “two” for two doses; and “boost” for booster dose.

## DISCUSSION

A higher proportion of vaccinated people was identified among female, elderly people aged 80 or over, those of Asian race/skin color, those in the Southeast region, those who did not receive respiratory support and those who did not die. The vaccination schedule, especially administration of the booster dose, proved to be effective in increasing survival for all age groups in the sample studied.

In 2021, a large number of unimmunized cases were noted, which contributed to the increase in mortality in the population evaluated. The National Immunization Program (*Programa Nacional de Imunizações* - PNI), important for reducing cases and deaths due to various vaccine-preventable diseases ever since its creation, lost its lead role in conducting the vaccination campaign against COVID-19, due to political-ideological interests.^16^ The slowness in starting the vaccination campaign delayed 316 million doses that could have been administered in 2021, a number sufficient to vaccinate 78% of the population and prevent the deaths of a considerable number of Brazilians affected by COVID-19.^17^

Our analysis showed a higher percentage of vaccinated female. Women, historically, seek health services and care more than men.^18^ There are several reasons that influence these gender inequalities in access to health services. There is a belief rooted in society that women seek health services more, as they are more concerned, interested, fragile and susceptible to diseases,^19^ making them seek care more. On this point, it is worth mentioning that the first person to be vaccinated in Brazil was a Black female public health service nurse in the state of São Paulo, a fact that was repeated in several Brazilian states.^20^ Her attitude can have served as an incentive for other women to seek immunization.

A higher percentage of vaccinated people was found among the elderly, especially those who had received a booster dose. This result was expected, since it is the first priority age group for vaccination in Brazil. Furthermore, survival was higher among those vaccinated with two doses or a booster dose as well. Similar results were found in a study on COVID-19 mortality and vaccination coverage in China conducted at the beginning of 2022, in which risk of death was higher among the elderly; however, that risk was reduced through vaccination, especially with more than one dose.^21^ Another study, developed in Sweden, on mortality among residents of long-term care institutions in 2022, showed that a fourth dose of vaccine would imply a reduction in the risk of death among the elderly, when compared to risk of death for those who received only three doses.^22^

When looking at the Brazilian geographic macro-regions, the Southeast had the highest percentage of vaccinated people, especially with the booster dose. It was in this region where vaccination began in Brazil, on January 17, 2021, hours after the National Health Surveillance Agency (*Agência Nacional de Vigilância Sanitária* - Anvisa) approved the emergency use of CoronaVac and AstraZeneca immunizers: the São Paulo government was the first to vaccinate against COVID-19, prioritizing health professionals, Indigenous people and *Quilombolas* in that state.^23^ Brazil’s vaccine production hubs are in the Southeast region, where the Butantan Institute, in the São Paulo state capital, and Fiocruz in the city of Rio de Janeiro, are located. Both institutions are responsible for supplying 70% of the Brazilian public sector demand for immunization agents. It was precisely the partnership agreement signed between the São Paulo state government and Sinovac Biotech, an important biopharmaceutical company in China, that enabled production of CoronaVac by the Butantan Institute.^24^

The Northern region of Brazil had the lowest percentage of vaccinated people. This region has the largest number of Indigenous people, a priority group for vaccination in Brazil. This is possibly due to the concentration of Primary Health Care (PHC) teams, mainly in the states of Amazonas and Acre, on the banks of large rivers, leaving the population furthest away from these river courses uncovered. In states such as Pará, Rondônia and Tocantins, there is a greater presence of teams in cities distributed along highways.^25^ Differences in access to and use of health services can be seen between the country’s macro-regions: the North and Northeast have the poorest assessment of health status and lower use of services, despite these regions having greater public program coverage.^26^

The results of this study showed that the vaccination schedule, especially the booster dose, increased the survival of the entire sample, in different age groups. A systematic review with meta-analysis, on data from several countries, showed that vaccination against COVID-19 reduces the risk of presenting a severe form of the disease, regardless of the laboratory that manufactures the vaccine product.^27^ Another systematic review, on studies with data from the United States and the United Kingdom, adds that administering the vaccine reduces the rate of infection, hospitalization and mortality among different populations, with Pfizer/BioNTech® being the most effective against infections caused by the B.1.1.7 and B.1.351 variants of SARS-CoV-2.^28^ Furthermore, two doses of vaccine increase immunization when compared to just one dose.^29^

A limitation of this study is the incompleteness of some variables included in the analyses and, in order to minimize this problem, the imputation method was used for variables with missing information. Despite this limitation, the study used the largest SARS database in Brazil. It has reliable information and has proven to be homogeneous in capturing and disseminating data,^30^ which allows inferences to be drawn from vaccination information for the rest of the country. Furthermore, to minimize the effects of errors in reporting vaccination data, only cases integrated into the National Health Data Network (*Rede Nacional de Dados em Saúde* - RNDS) were used, which provides information on vaccine doses with greater security.

The results of this study suggest that vaccination against COVID-19 increased survival and reduced the risk of death among people who presented a severe or critical form of the disease, regardless of age group. They also show that the complete vaccination schedule, especially with a booster dose, provides more protection to these individuals, especially the elderly. Therefore, the need for continuous and broad vaccination of the entire population is emphasized, with regular boosters in different population groups.

## References

[1] Duan Y, Shi J, Wang Z, Zhou S, Jin Y, Zheng Z (2021). Disparities in COVID-19 Vaccination among Low-, Middle-, and High-Income Countries: The Mediating Role of Vaccination Policy. Vaccines.

[2] Leonel F (2022). Brasil celebra um ano da vacina contra a Covid-19.

[3] Bastos LS, Ranzani OT, Souza TML, Hamacher S, Bozza FA (2021). COVID-19 hospital admissions: Brazil’s first and second waves compared. Lancet Respir Med.

[4] Pifano SLA, Ferreira CMSD, Miranda AMVM, Xavier BB, Almeida BS, Barcelos CSM (2022). Impacto da vacinação em massa de trabalhadores da saúde no afastamento de suas atividades laborais pela covid 19 em um hospital terciário. Braz J Infect Dis.

[5] Brasil (2022). Vacinas - Covid-19.

[6] Lana RM, Freitas LP, Codeço CT, Pacheco AG, Carvalho LMF, Villela DAM (2021). Identification of priority groups for COVID-19 vaccination in Brazil. Cad Saude Publica.

[7] Lilla JAC, Amaral AC, Tranchesi RAM, Mansur NS, Laranjeira R, Medeirosa EAS (2022). Impacto da vacinação e das medidas de prevenção para covid-19 em trabalhadores da área da saúde de 12 hospitais do estado de São Paulo. Braz J Infect Dis.

[8] Castro R (2022). Vacinas contra a Covid-19: o fim da pandemia?. Physis.

[9] Hardgrave H, Wells A, Nigh J, Klutts G, Krinock D, Osborn T (2022). COVID-19 Mortality in Vaccinated vs. Unvaccinated Liver & Kidney Transplant Recipients: A Single-Center United States Propensity Score Matching Study on Historical Data. Vaccines.

[10] Ahmed S, Mehta P, Paul A, Anu S, Cherian S, Shenoy V (2022). Postvaccination antibody titres predict protection against COVID-19 in patients with autoimmune diseases: survival analysis in a prospective cohort. Ann Rheum Dis.

[11] Gyeltshen K, Tsheten T, Dorji S, Pelzang T, Wangdi K (2021). Survival Analysis of Symptomatic COVID-19 in Phuentsholing Municipality, Bhutan. Int J Environ Res Public Health.

[12] Ramos RH, Ferreira COL, Simão A The Survival Rate Among Unvaccinated, First Dose, and Second Dose Brazilian Hospitalized and ICU COVID Patients by Age Group.

[13] Sales-Moioli AIL, Galvão-Lima LJ, Pinto TKB, Cardoso PH, Silva RD, Fernandes F (2022). Effectiveness of COVID-19 Vaccination on Reduction of Hospitalizations and Deaths in Elderly Patients in Rio Grande do Norte, Brazil. Int J Environ Res Public Health.

[14] Santos CVBD, Noronha TG, Werneck GL, Struchiner CJ, Villela DAM (2023). Estimated COVID-19 severe cases and deaths averted in the first year of the vaccination campaign in Brazil: A retrospective observational study. Lancet Reg Health Am.

[15] Buuren S van, Groothuis-Oudshoorn K (2011). Mice: Multivariate Imputation by Chained Equations in R. J Stat Softw.

[16] Maciel E, Fernandez M, Calife K, Garrett D, Domingues C, Kerr L (2022). A campanha de vacinação contra o SARS-CoV-2 no Brasil e a invisibilidade das evidências científicas. Cien Saude Colet.

[17] Hallal PC (2021). SOS Brazil: science under attack. Lancet.

[18] Cobo B, Cruz C, Dick PC (2021). Gender and racial inequalities in the access to and the use of Brazilian health services. Cien Saude Colet.

[19] Carneiro VSM, Adjuto RNP, Alves KAP (2019). Saúde do homem: identificação e análise dos fatores relacionados à procura, ou não, dos serviços de atenção primária. Arq ciências saúde UNIPAR.

[20] Fernandes CM, Farnese P, Garcia JM, Demuru P (2021). Imunização e desigualdade de gênero: a construção da imagem da mulher nos primeiros atos de vacinação contra a covid-19. Revista Eletrônica de Comunicação, Informação & Inovação em Saúde.

[21] Smith DJ, Hakim AJ, Leung GM, Xu W, Schluter WW, Novak RT (2022). COVID-19 Mortality and Vaccine Coverage — Hong Kong Special Administrative Region, China, January 6, 2022–March 21, 2022. MMWR Morb Mortal Wkly Rep.

[22] Nordström P, Ballin M, Nordström A (2022). Effectiveness of a fourth dose of mRNA COVID-19 vaccine against all-cause mortality in long-term care facility residents and in the oldest old: A nationwide, retrospective cohort study in Sweden. Lancet Reg Health Eur.

[23] Bitar R (2022). Há um ano, SP vacinava 1a pessoa contra Covid no Brasil; veja o que mudou e projeções para o futuro.

[24] Pazelli GS, Chudzinski-Tavassi AM, Vasconcellos AG (2022). Desenvolvimento de Vacinas: o potencial do Instituto Butantan na Pandemia de Covid-19. Cadernos de Prospecção.

[25] Garnelo L, Lima JG, Soares E (2018). Access and coverage of Primary Health Care for rural and urban populations in the northern region of Brazil. Saúde debate.

[26] Viacava F, Bellido JG (2016). Health, access to services and sources of payment, according to household surveys. Cien Saude Colet.

[27] Huang YZ, Kuan CC (2022). Vaccination to reduce severe COVID-19 and mortality in COVID-19 patients: a systematic review and meta-analysis. Eur Rev Med Pharmacol Sci.

[28] Mohammed I, Nauman A, Paul P, Ganesan S, Chen K, Jalil SMS (2022). The efficacy and effectiveness of the COVID-19 vaccines in reducing infection, severity, hospitalization, and mortality: a systematic review. Hum Vaccin Immunother.

[29] Chodick G, Tene L, Rotem RS, Patalon T, Gazit S, Ben-Tov A (2022). The Effectiveness of the Two-Dose BNT162b2 Vaccine: Analysis of Real-World Data. Clin Infect Dis.

[30] Silva GA, Jardim BC, Lotufo PA (2021). Mortalidade por COVID-19 padronizada por idade nas capitais das diferentes regiões do Brasil. Cad. Saúde Pública.

